# Comfort and vibration level of children in cycle carriers

**DOI:** 10.1371/journal.pone.0282778

**Published:** 2023-03-09

**Authors:** Malte Rothhämel

**Affiliations:** 1 KTH Vehicle Dynamics, Royal Institute of Technology, Stockholm, Sweden; 2 Center for ECO^2^ Vehicle Design, Royal Institute of Technology, Stockholm, Sweden; Al Mansour University College-Baghdad-Iraq, IRAQ

## Abstract

This paper presents an analysis of comfort measurements taken in a bicycle trailer for transport of children. The vibration level was then compared to that in a cargo-trike and in a passenger car. The current research contributes to the rare literature on passenger comfort in bicycle trailers through measurements with accelerometer sensors between the seat in the trailer and a dummy representing an infant child. The varied parameters were the tyre inflation pressure, the driving velocity, as well as the additional load in the trailer. The results show a quite high weighted acceleration of 1...3ms2 on asphalt and 3...5ms2 on cobblestone pavement, which is similar to that in a compared cargo-trike but significant higher than the vibration level in the compared car.

## Introduction

Cycling is a well-established fossil-free mode of transport with a minimum of exhaust gas and noise emissions as well as practically free of toxic emissions and particle matter. An increase of sustainable and active transportation is one of the goals more or less all larger cities in the world have formulated [1, 2]. Everyday cycling can be utilitarian when deemed as an efficient, comfortable, and sustainable mode of transport. However, cyclists are as other road users dependent on infrastructure as roads.

This paper presents an analysis of comfort measurements taken in a bicycle trailer for transport of children. The current research contributes to the rare literature on passenger comfort in bicycle trailers through measurements with accelerometer sensors between the seat in the trailer and a dummy representing an infant child. The varied parameters were the tyre inflation pressure, the driving velocity, as well as the additional load in the trailer.

Studying bicycle and bicycle passenger comfort is an important research topic for at least three reasons. First, it is closely related to sustainable mobility, as the problem of low-emission and (sub-)urban mobility is often planned to be solved through an increased use of bicycles. Second, bicycle as mode of transportation is more and more in focus even for simulation tools supporting planning of infrastructure. So, there is need for empirical data for the simulation of bicycles as means of transport. Third, depending on the level of discomfort, the dose of vibration can rise to a question of health as it already was the case in other branches of transport as Grandlund showed for heavy trucks [3] and Rehnberg and Drugge for wheel loaders [4]. Hull [5] summarises this as comfort goes hand in hand with safety and “identifies one of the barriers to encouraging more cycling is the inexperienced cyclist’s perception of the safety, comfort and continuity of the cycling network in their city”.

It is a general question in the larger context of active transport: Cycling to work is in principal recommended for personal health [6] and—since associated with less absenteeism [7]—also of national economic importance. From cyclists it is broadly known that a smooth road surface is of high priority for the route choice [8, 9]. However, longer routes increase the threshold to use the bicycle for utility trips at all. Generally, the experienced quality of service is directly affected by the pavement-surface conditions [10]. The university of Hannover has therefore initiated the project Ridevibes where utility cyclists use a routing app on a mobile device that takes comfort as function of pavement-surface conditions into account. At the same time the users collect data with their mobile device about exactly these conditions they experience [11].

In automobile development, comfort is an important aspect since many decades back but still an actual research topic: Yang et al. [12] compared the control of a semi-active suspension system on a research car with and without a LIDAR preview. At a vehicle speed of 20ms over an arced obstacle of 60 cm length and 8 cm height they could reduce the RMS value of vertical acceleration from 3.2ms2 to 2.7ms2. With a different approach Lantoine et al. [13] compared three different seats whereof on had an own suspension. By means of installed pressure mats they could measure the pressure and its distribution between seat and body. In addition, they interviewed the subjects during driving about their experienced level of discomfort. They found that the seat with suspension caused minor local discomfort. Anyhow, on all three car seats the discomfort over time grew similarly.

Taking the reasons into account why people do not chose the bicycle as natural mode of transport [14], Dickinson et al. [15] found that “some factors are more important for specific groups. Women, in particular, cite the difficulty of combining a journey with picking up children or shopping as a reason for not cycling.” This shows that the applicableness of cargo bicycles or trailers is of high importance.

Comfort measurements on bicycles have been performed e.g. by Hölzel et al. [16] comparing asphalt, concrete slabs, self-binding gravel and cobblestones where the comfort decreased in this order and with increasing bicycle speed.

Hastings et al. [17] tested how the human power of a professional rider on three different road racer bikes on a treadmill was affected where the roller was equipped with an unevenness. However, there was no evidence for a reduced human power caused by the disturbance.

The Swedish National Road and Transport Research Institute (VTI) developed and conducted experiments to assess the riding quality perceived by cyclists based on the longitudinal profile of a cycle path [18]. One of the tests comprised a step with varying height where 5 mm was hardly experienced, 8-10 mm was recognisable and 12 mm was clear uncomfortable.

Nilimaa [19] found a significant correlation between driving vibrations and megatexture (50 mm…500 mm wavelength). For shorter wavelengths the relation was not that clear.

The experiment closest to the presented study was published by Schwanitz et al. [20]. They tested the vibration level in a bicycle trailer for child transport with two dummies of different size on different road surfaces and measured the vibration level and investigated the influence factors by means of an analysis of variance.

Moreover, a very comprehensive master thesis work was done by van Driesschen [21]. He investigated a suspension system made to mount a baby car seat in a cargotrike and compared it to measured accelerations levels in cars. The main part of the work is the design of an improved seat suspension version with better comfort properties. With that he reduced the vertical acceleration level by about 50%.

The study by Schwanitz et al. was used as baseline for the presented study. However, the scope was to expand the range, on the one hand by means of another range of tyre pressure. In the cyclists’ community tyre pressure and comfort is always a topic for discussions. On the other hand, another impact was meant to be tested. I virtual “curb” was realised by means of a clear defined step in the height as it occurs in reality on bicycle infrastructure. In addition, another type of child transport was integrated in the experiments to benchmark not only against an automobile but also within the cycling domain. Finally, the evaluation of the the frequencies was planned in a similar way van Driesschen did. Human subjects were not considered. On the one hand the advantage of questioning as Lantoine et al. [13] did in their automobile car seat study, would not be present in experiments with infants or toddlers anyhow. On the other hand there might be ethical concerns even if only measurements within reasonable parameter ranges were considered.

## Theory

Vibration often occurs in form of sinusoidal movements of parts. These vibrating parts can excite the air resulting in sound or excite a human body in contact. The human skin detects vibrations mainly by means of Meissner’s and Pacinian corpuscles. Disturbing vibrations can be annoying or even harmful to health. Anyhow, maximum permissible values are currently only available in the area of occupational health and safety, see ISO 2631 [22]. According to the author’s knowledge, specifically for children, legal boundary values do not exist.

Since vibration usually is a sequence of superposed frequencies and amplitudes over time, it is necessary to reduce this time series to a value enabling comparisons. So, in this work the weighted vibration level was used for continuous measurements, while the fourth power vibration dose (VDV) was used for the characterisation of short term impacts. Both of these values are suggested in the ISO 2631 and were calculated accordingly.

While VDV and weighted vibration level compress data, vibrations can be fragmented for analysis. In this work Fast Fourier Transformation (FFT) was used to compare the portions of frequencies in the spectrum, see subsection *FFT* in section *Results*.

## Method

### Vehicles

**Fig 1:**
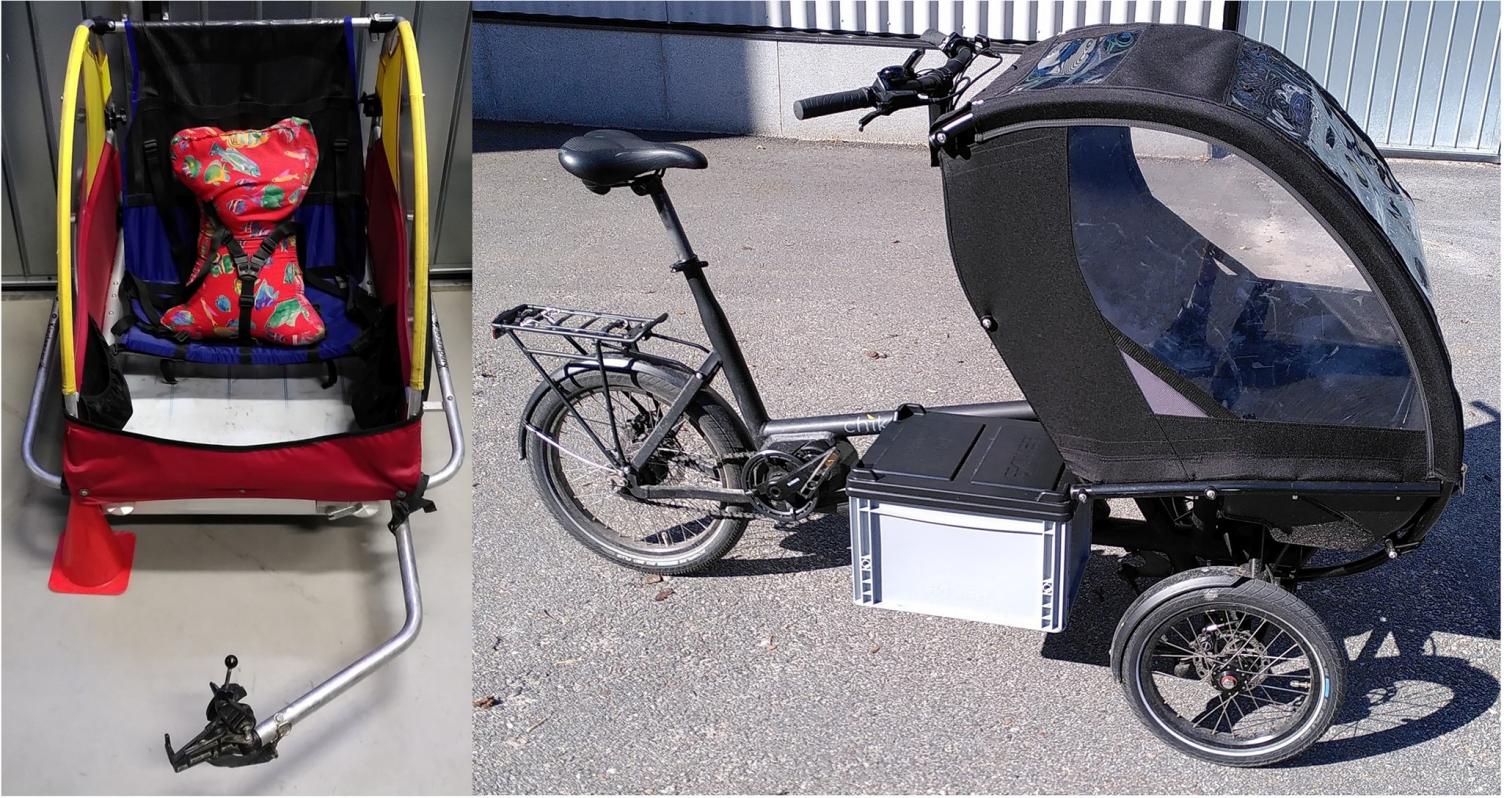
Test vehicles for child transport used in this study: Cycle carrier Kindercar with dummy (left) and Cargotrike Chike.

The main test object was an older version of a bicycle carrier for the transport of one or two children made by the manufacturer Kindercar, see Fig 1 on the left. The trailer has an aluminium floor pan, seats made of tighten textile and rolls on 20 inch wheels with balloon tyres (Schwalbe Big Apple in ERTRO size 60-406 (ERTRO = European Tyre and Rim Technical Organisation: www.ertro.org)). There was no chassis suspension on this trailer. The maximum payload in the trailer is 60 kg. Its empty weight is 16.5 kg.

A second vehicle for comparison was a new cargo tricycle for the transport of one or two children made by the manufacturer Chike, see Fig 1 on the right. The tricycle is an electric assisted bicycle in the rear and two wheels in the front (Tadpole). The front wheels are lead by a double wishbones with an elastomer suspension that connects left and right side in a tilting mechanism. In front of the rider above the front wheels there is a cabin made of a plastic pan and a textile roof for up to two children. The maximum load in the cabin is 60 kg. The tyres mounted in the front were Schwalbe Big Apple in ERTRO size 50-305. In contrast to the bicycle carrier the trike includes in operation always the weight of the adult rider.

Another vehicle used for comparative measurements was a fully electric Volkswagen Golf from 2015. The vehicle was chosen as a representative of an average car but avoiding potential vibration from an internal combustion engine.

### Passengers / load

To avoid any ethical conflicts the experiments were conducted with dummies. A dummy was created with a cotton textile shell filled with oat flakes to simulate a yielding skin. The meaning was to take damping effects of the skin of a human being into consideration. The dummy had a weight of 6.5 kg, representing a infant in the age between 2 and 9 months [23]. Additional load in the trailer should be realistic and practical in handling. Additional weight mounted with screws or similar fastening was discarded as unrealistic in daily use. Therefore, juice in 1-litre-beverage cartons was used as representative additional load.

### Road surfaces and step input

**Fig 2:**
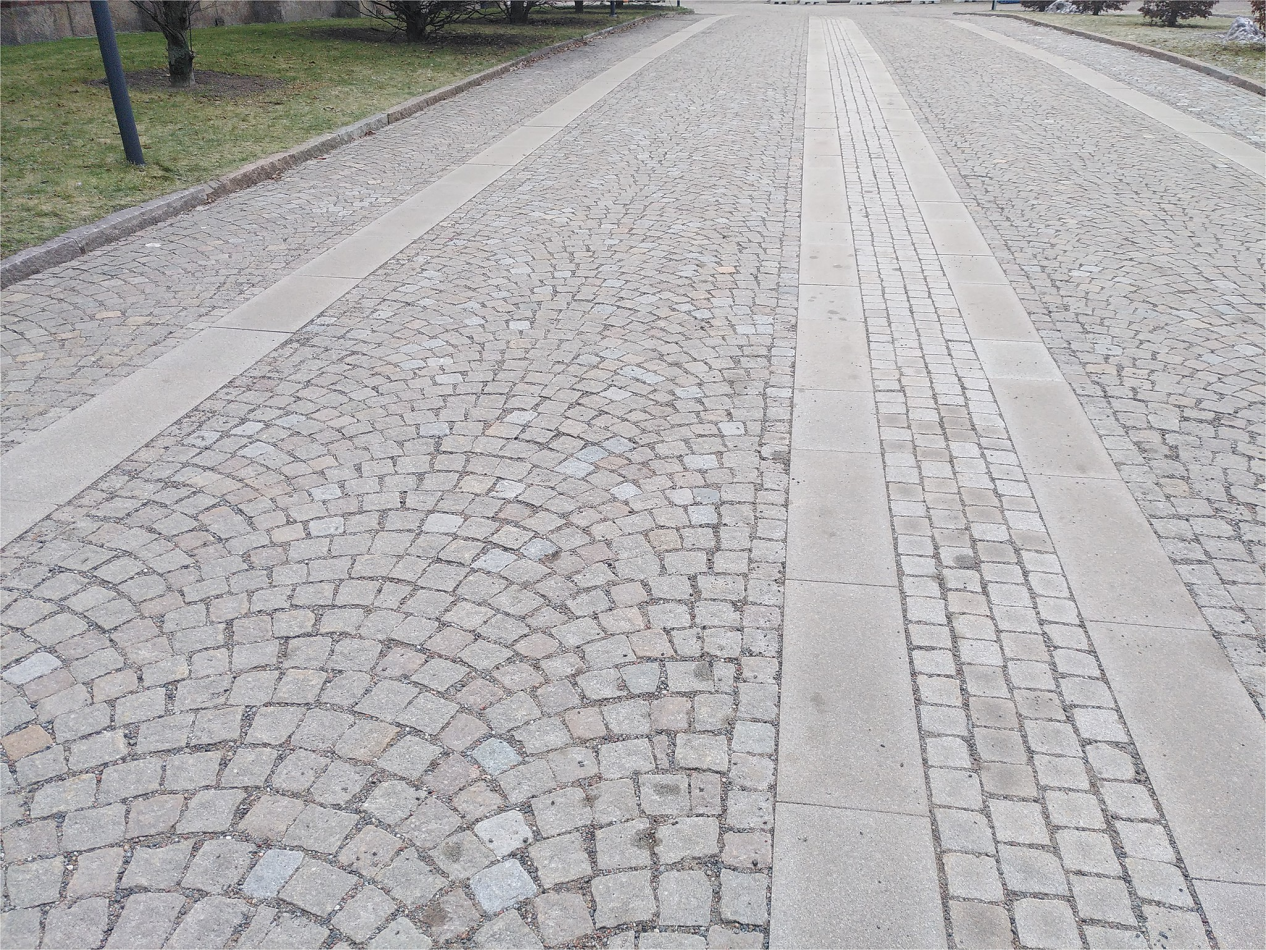
Cobblestone road surface for outdoor tests. The tests were carried out between the concrete slabs.

The outdoor experiments were conducted on the one hand on a cobblestone road surface (Fig 2) with small and relatively even laid stones to represent rough surfaces (feed size of the stones ∼ 8 cm). On the other hand a relative new asphalt surface was chosen to represent a fine surfaces.

**Fig 3:**
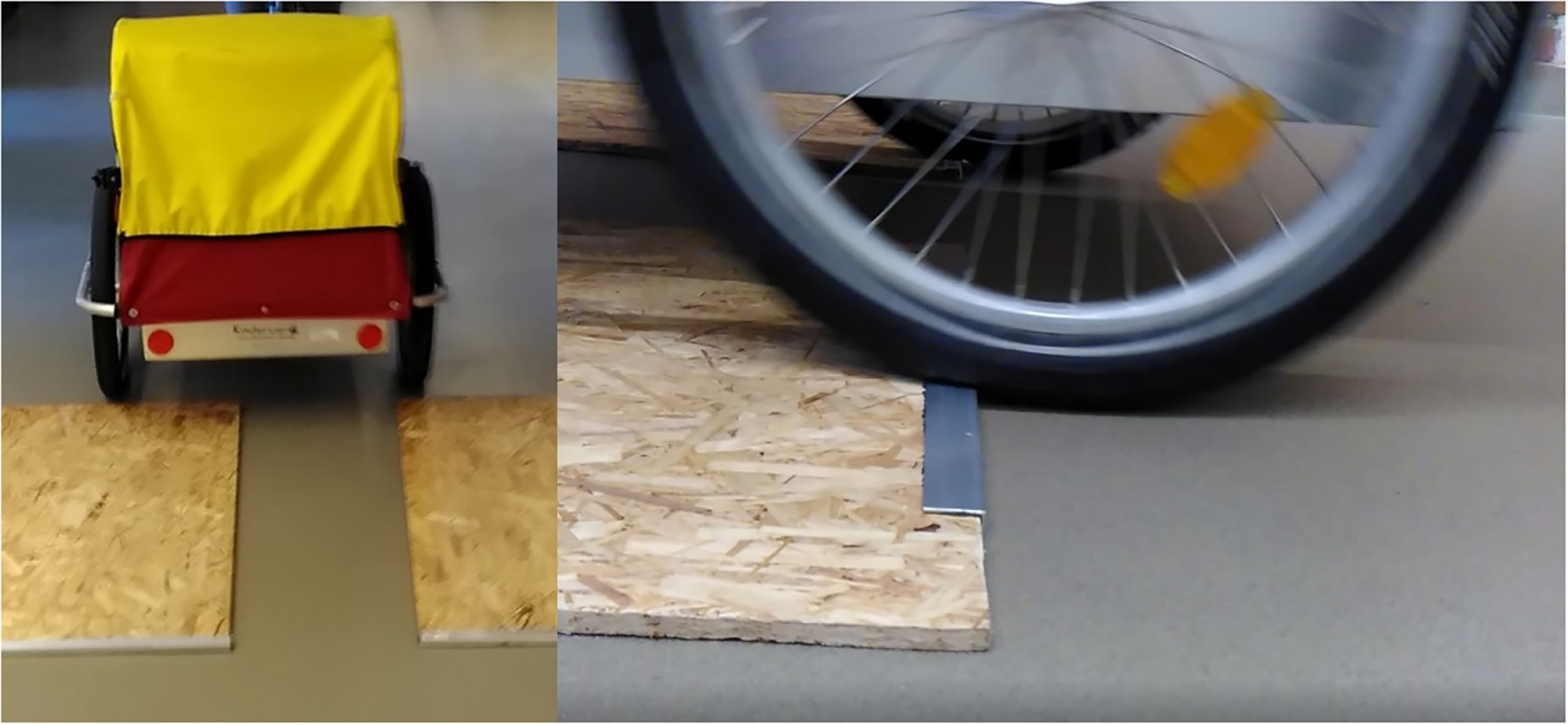
Picture of the step input utilised during the indoor-experiments. Cycle carrier running over two oriented strand boards with with a gap for the pulling bicycle (left). Cycle carrier wheel running over an oriented strand board with aluminium reinforced edge (right).

The indoor experiments were conducted on painted industrial concrete in a basement corridor. To create a repeatable unevenness, two oriented strand boards (OSB) were used and reinforced with an aluminium corner to avoid wear. Two boards were used next to each other (with a gap in-between for the pulling bicycle), which resulted in a step height of 14 mm, see Fig 3. Two additional boards without reinforced edge were used to increase the heigh up to 25 mm in total.

### Parameter variation

The parameters varied under the experiments were

Tyreinflationpressure25;100;300kPaRoadsurface(seesectionabove)Additionalload0;24kgVehiclespeed10;15;20kmh

The complete test plan for the cycle carrier can be seen in Tables 1 and 2. In the experiments conducted by Schwanitz et al. [20] the tyre inflation pressure hardly affected the results. However, the pressure level (300…500 kPa) was within the specification but, anyhow, quite high. Therefore, a lower range of tyre inflation pressure was chosen here. The tyre had an inflation pressure specification of 150…400 kPa at a maximum static load of 85 kg each. Since the weight of a carrier for child transport is much lower, a reduced minimum pressure can be technically accepted. Accordingly, the manufacturer of the carrier used in this experiment recommends 200 kPa tyre inflation pressure. The lower limit of 25 kPa was identified by means of a pre-test where a minimum possible pressure level was identified by means of some iterations in a static experiment. The additional load was determined according to the maximum possible weight that a grocery shopping fitting in the luggage space of the carrier, could take on. Castro [24] found that the average speed of cyclists on flat terrains ranges between 17kmh and 25kmh. It can be assumed that cyclists are slower with a trailer, specifically, when transporting children. Upper and lower representative cruising speeds were estimated at 10kmh and 20kmh respectively.

**Table 1:** RMS values for outdoor tests with child carrier.

Surface	Speed [kmh]	Infl. pr. [kPa]	Weight [kg]	*a*_*xyz*_ RMS mean [ms2]	*a*_*xyz*_ RMS std. dev. [ms2]
Asphalt	10	25	23	1.15	0.044
Asphalt	10	25	47	1.60	0.812
Asphalt	15	25	23	1.95	0.277
Asphalt	15	25	47	1.79	0.145
Asphalt	20	25	23	3.12	0.174
Asphalt	20	25	47	2.97	0.405
Asphalt	10	100	23	1.38	0.023
Asphalt	10	100	47	1.28	0.083
Asphalt	15	100	23	1.90	0.052
Asphalt	15	100	47	2.05	0.124
Asphalt	20	100	23	3.28	0.116
Asphalt	20	100	47	4.36	0.379
Asphalt	10	300	23	1.40	0.051
Asphalt	15	300	23	2.01	0.069
Asphalt	20	300	23	2.92	0.174
Cobblestones	10	25	23	2.84	0.099
Cobblestones	10	25	47	3.40	0.503
Cobblestones	15	25	23	3.82	0.259
Cobblestones	15	25	47	3.07	0.310
Cobblestones	20	25	23	4.77	0.370
Cobblestones	10	100	23	4.25	0.115
Cobblestones	10	100	47	6.16	0.191
Cobblestones	15	100	23	4.88	0.180
Cobblestones	15	100	47	9.12	0.572
Cobblestones	20	100	23	5.42	1.043
Cobblestones	20	100	47	10.16	0.603
Cobblestones	10	300	23	5.50	0.093
Cobblestones	15	300	23	6.13	0.120
Cobblestones	20	300	23	6.94	0.190

**Table 2:** RMS values for indoor tests over the step input (threshold height 14 resp. 25 mm) with child carrier.

Threshold mm	Speed [kmh]	Infl. pr. [kPa]	Weight [kg]	*a*_*xyz*_ RMS mean [ms2]	*a*_*xyz*_ RMS std. dev. [ms2]
14	10	300	23	3.64	0.109
25	10	300	23	4.86	0.221
14	20	300	23	4.83	0.154
25	20	300	23	6.35	0^1)^
14	20	25	23	3.72	0.188
25	20	25	23	5.21	0.206
14	10	25	23	3.15	0.138
25	10	25	23	4.12	0.127

^1)^ In this test run only one measurement was available, most of the data was unusable because of a loose cable.

### Test operation

The dummy was situated in the center of the seats as supposed to for single children transport. The acceleration sensor was positioned under the dummy between dummy and seat. The trailer was attached to a bicycle and a cable-switch drawn to the handlebar. When reaching steady-state conditions on the test surface, the measurement was started by means of the switch. The vehicle speed was maintained as constant as possible manually by the rider. A bicycle computer provided speed information. For each setting of parameters five repetitions in each direction over the test surface were done, resulting in 10 measurements for each setting. All tests were performed in a temperature range of 15 to 25°C to assure a constant tyre behaviour.

### Measurement equipment

For the outdoor tests an ADXL345 3-axis acceleration sensor from Analog Devices (Output data rate: 800 Hz; Max. operational level (peak): 16 g; Resonance freq.: *not specified*) was used and connected via an I^2^C interface with an Adafruit M0 feather that saved the data on a micro SD card. With this setting the sampling frequency was limited to 56 Hz. For the indoor tests (step input) three accelerometers of type 4349 from Brüel & Kjaer (Freq. range: 1—25000 Hz; Max. operational level (peak): 500 g; Resonance freq.: 52 kHz) were used in combination with a National Instruments 9234 data acquisition system. The sampling frequency was set to 2048 Hz. The sensors were with the respective DAQ calibrated on a steady-state 1 g-calibrator in the beginning of the measuring campaign.

### Vibration analysis

The tyres are the most elastic parts in the chain of vertical load transfer. And the tyre parameter affecting the vertical stiffness of the tyres mostly, is inflation pressure. So, the modification in tyre inflation pressure is assumed to have the largest effect on the frequency of the trailer behaviour. To assess this, the measurements were analysed by means of weighted vibration level and VDV according to ISO 2631 and by means of Fast Fourier Transformation (FFT). The results for similar experiments (same parameter setting) were averaged. For the evaluation of the outdoor experiments by means of FFT a section of 17.5 s was taken from the center of each steady-state measurement. To evaluate the indoor experiments a section of 2 s was taken symmetrically around the step-input, identified by means of the maximum vertical acceleration value. For comparison with previous research the unweighted root mean square (RMS) of the each driving was calculated. Since the seat is tilted it is hardly possible to guarantee a certain z-position. Therefore, the RMS calculated from measurements in this work is always a combination of all three directions.

## Results

In this section the results are presented from the outdoor tests with the trailer. For comparison, results are added from the measurements on the cargo-trike and in the car. This section is then completed with the indoor step-response measurements on the oriented strand boards.

### Outdoor experiments

**Fig 4:**
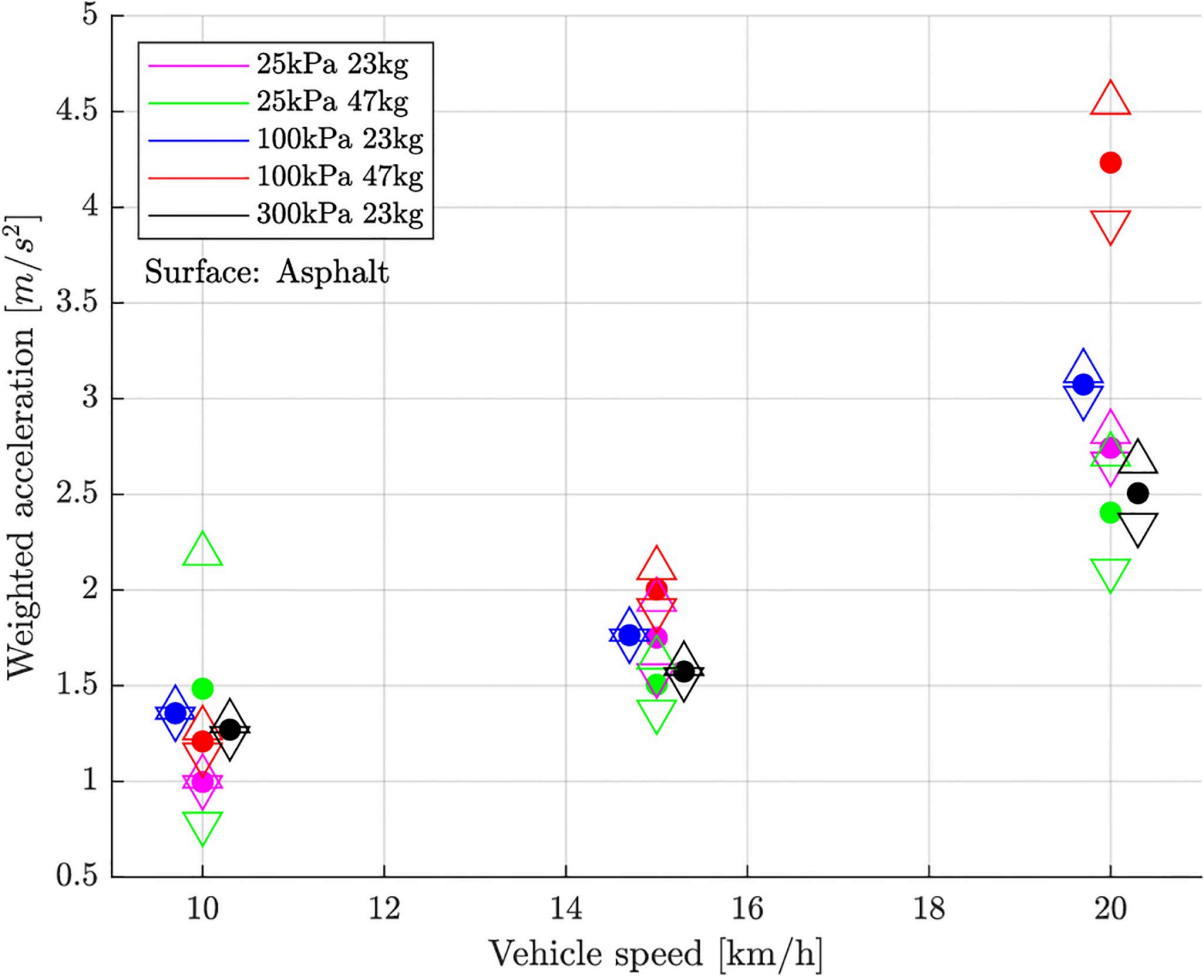
Child carrier: Weighted vibration level over vehicle speed on fine asphalt surface. △ and ▽ indicate the 95% confidence interval. Black and blue values have been moved laterally by a few mm for better visibility.

**Fig 5:**
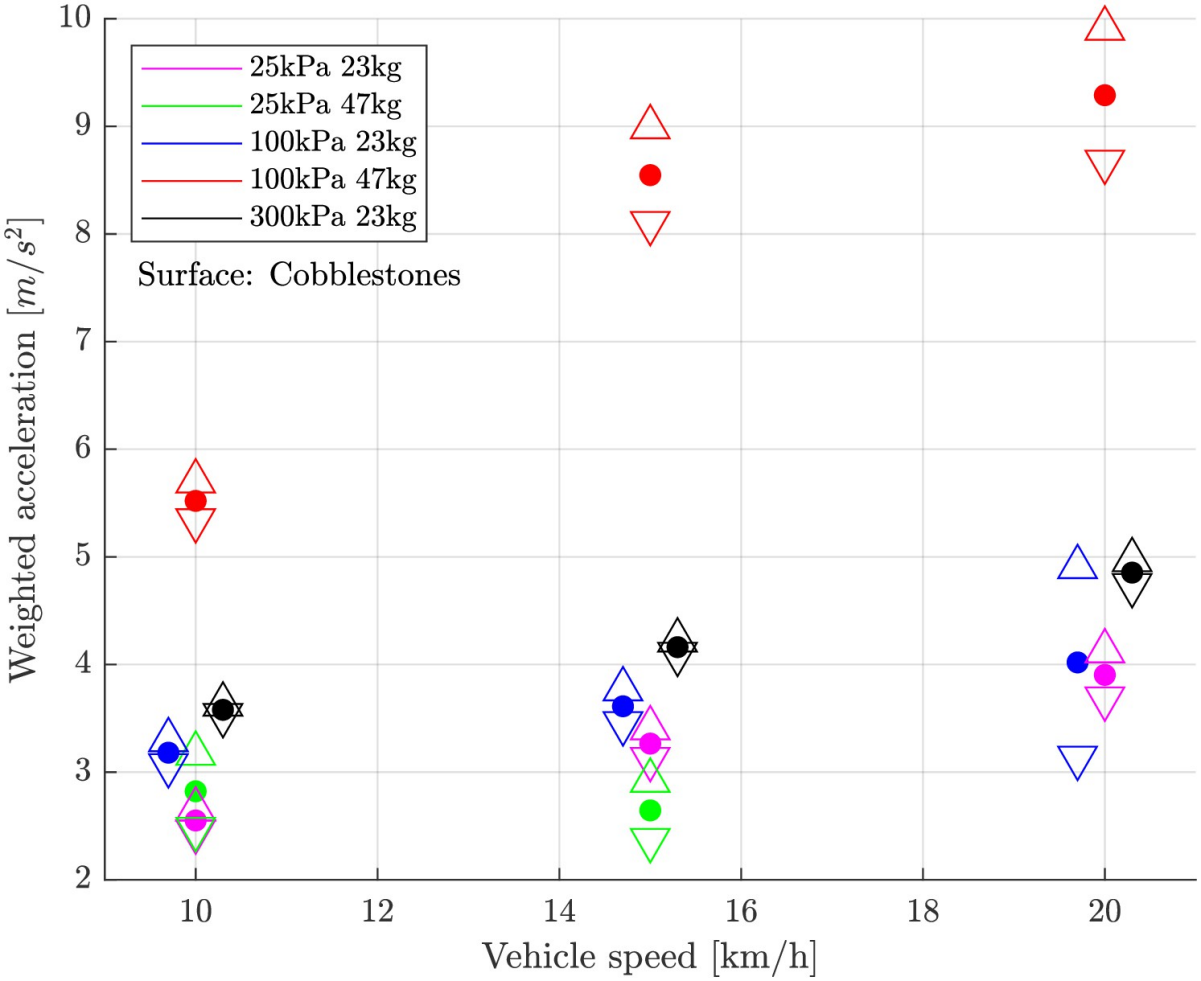
Child carrier: Weighted vibration level over vehicle speed on cobblestones surface. △ and ▽ indicate the 95% confidence interval. Black and blue values have been moved laterally by a few mm for better visibility.

The weighted vibration level increased with increasing vehicle speed (Fig 4). This is in general valid for both surfaces, asphalt and cobblestones (Fig 5). On asphalt the vehicle speed affects the weighted vibration level significantly more than tyre inflation pressure that can hardly be distinguished. On cobblestones the tyre inflation pressure has an influence of the same magnitude as the vehicle speed. Anyhow, the general vibration level is significant higher on cobblestones than on asphalt.

If an extra load of 24 kg was added in the carrier, the weighted vibration level increased at a tyre inflation pressure of 100 kPa. At extremely low tyre inflation pressure (25 kPa) the vibration level sank a little. At 20kmh and tyre inflation pressure 25 kPa data loss occurred. Measurements at tyre inflation pressure of 300 kPa and extra load were not conducted due to the already high vibration level at 100 kPa and extra load.

Table 1 shows then the mean of these RMS over all drivings with equal settings and the standard deviation of these RMS. In addition, this table also shows the complete parameter variation of the outdoor tests with the tested bicycle trailer.

Table 2 shows similarly the mean of the RMS over all drivings over the step input (for the height of the step see threshold value in the table) with equal settings and the standard deviation of these RMS. In addition, this table also shows the complete parameter variation of the indoor tests with the tested bicycle trailer.

### Comparison with trike and car

**Fig 6:**
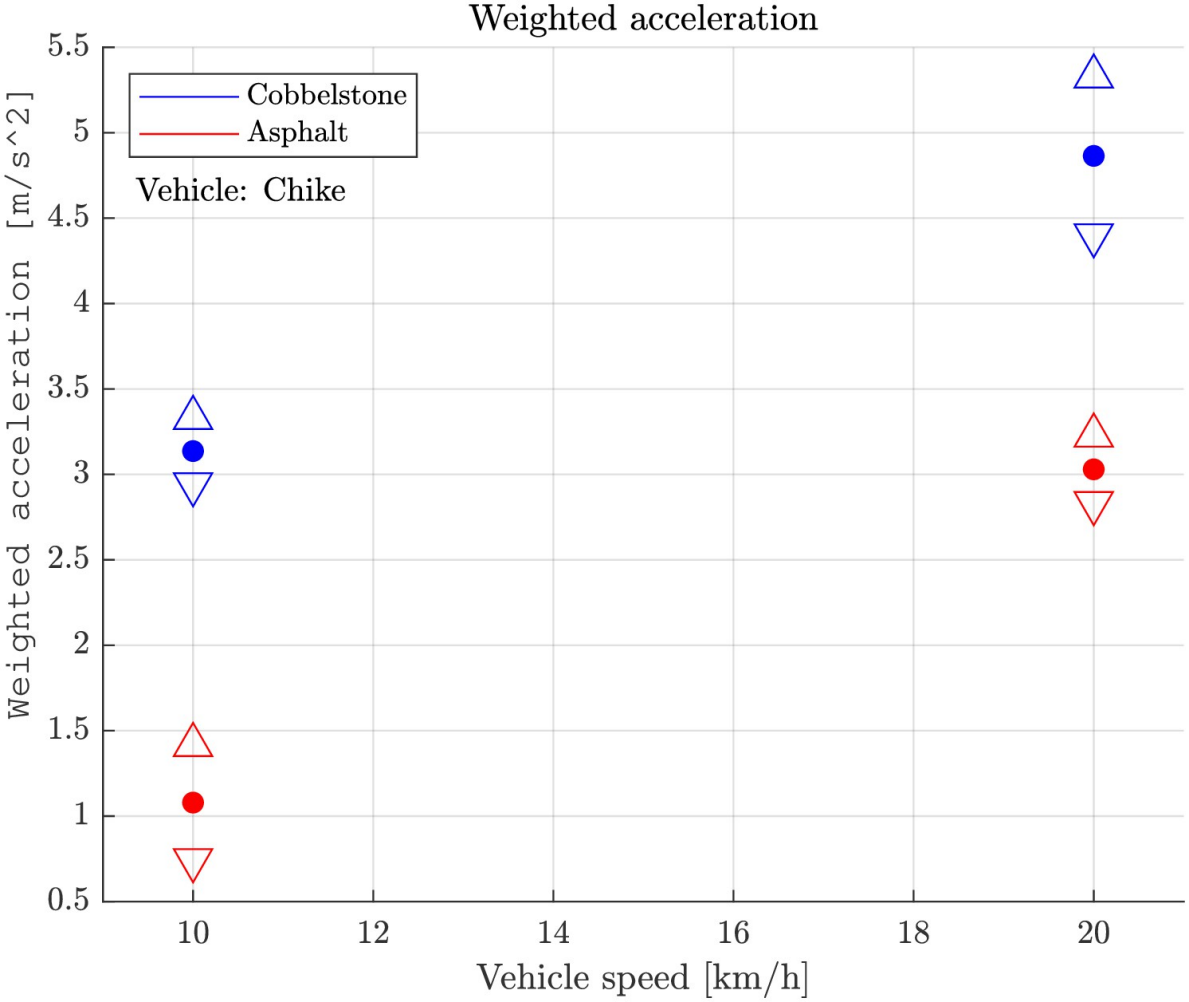
Cargo-Trike “Chike”: Weighted vibration level over vehicle speed on asphalt and cobblestones surface. △ and ▽ indicate the 95% confidence interval.

The cargo-trike was available for a few hours only, enabling some of the experiments. The tyre pressure was not modified, instead the recommended tyre pressure (300 kPa) was maintained. The weighted vibration level for the trike was in a similar magnitude as for the bicycle trailer for both on asphalt as well as on cobblestones (Fig 6).

On the same surfaces a set of measurements was taken in a car. Dummy and measuring equipment where identical. The dummy was placed in a car child seat on a rear seat. The vehicle speed was set to 30kmh and maintained by means of a cruise control function. The results show a significant lower value than in the bicycle transport devices, see Table 3. Tyre pressure was not modified because of the assumption that the chassis suspension has a larger effect than the tyre suspension. The vehicle speed was not either increased because of safety constraints in the test area. A lower vehicle speed was difficult to maintain constantly.

**Table 3:** Summarising acceleration values for compared automobile as unweighted RMS and weighted acceleration according to ISO 2631.

Surface	Vehicle speed [kmh]	Unweighted RMS [ms2]	Weighted vibration level [ms2]
Asphalt	30	1.01	0.77
Cobblestone	30	1.42	1.32

### Indoor experiments—Step response

**Fig 7:**
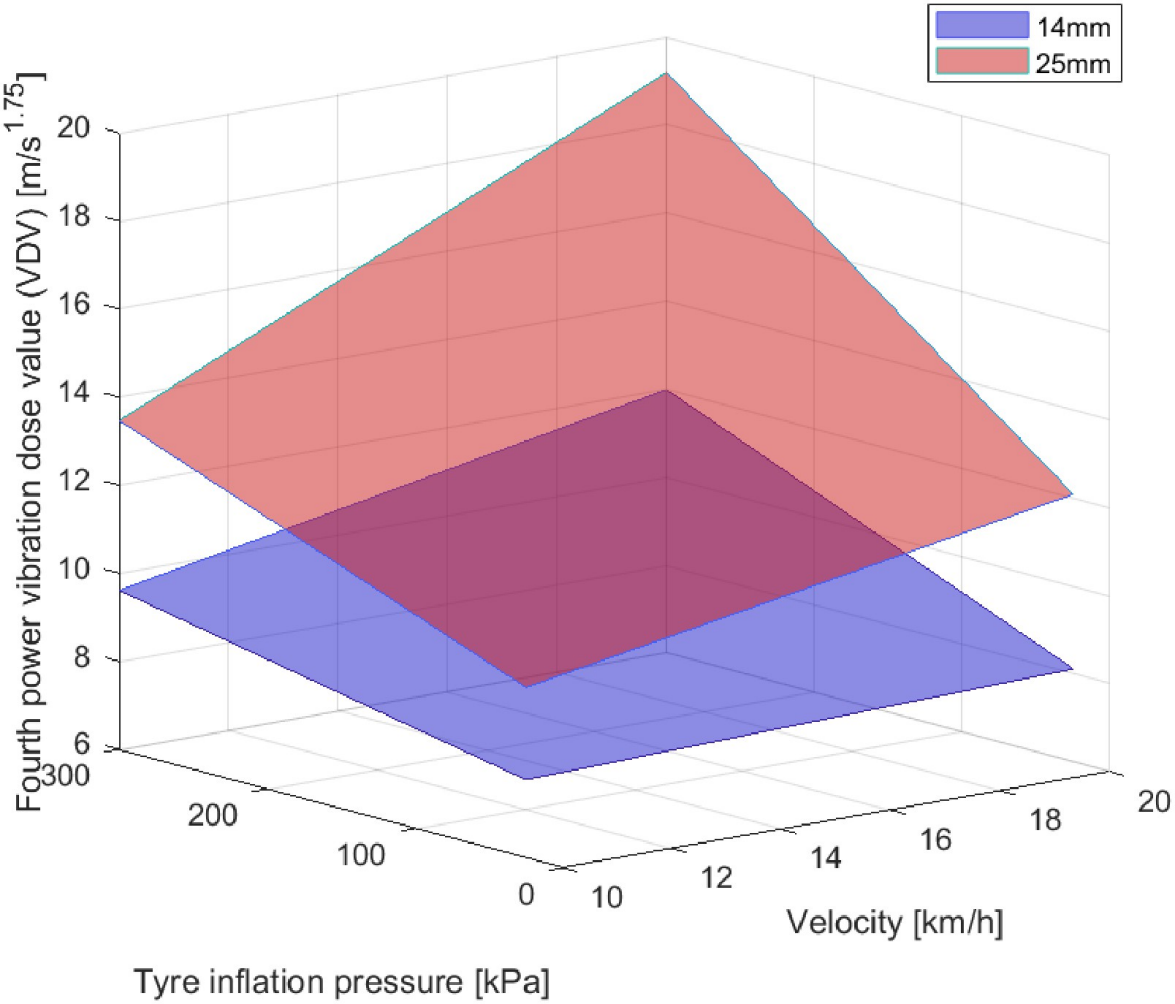
Fourth power vibration dose value (VDV) in accordance to ISO 2631 over tyre inflation pressure and vehicle speed. The graph visualises the measurement results of the bicycle trailer over a step (height 14mm resp. 25mm).

In an indoor experiment driving over a synthetic edge was investigated. In contrast to the before described experiments on road surfaces with different texture the curb is similar with each driving since the aluminium reinforced edge had a height of 14 mm resp. 25 mm. An uncertainty was given by the angle of attack. Depending on this angle there could occur a certain delay in the impact of the wheels on the edge. Since the impact is a single occasion, the evaluation must take this single event into consideration. According to ISO 2631 the fourth power vibration dose value (VDV) is an appropriate evaluation method. After the experience with additional load in the outdoor experiments, only vehicle speed, inflation pressure and road surface—meaning here the height of the step—were varied. The results are visualised in a 3D-plot (Fig 7). It becomes clear that VDV increases with both, threshold height, tyre inflation pressure and vehicle speed.

### FFT

**Fig 8:**
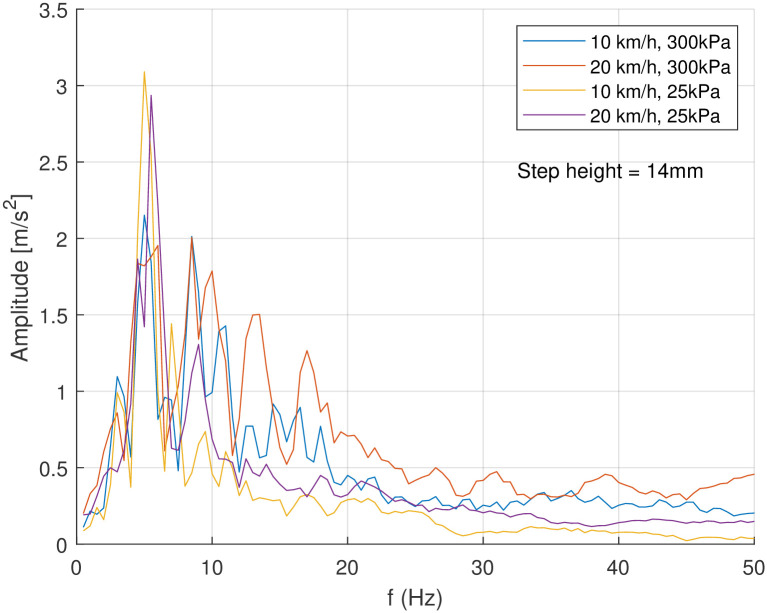
Single sided amplitude spectrum of the vertical acceleration *a*_*z*_ as result of an FFT when driving the bicycle trailer over a step (14 mm). For each setting about ten test runs were combined by means of arithmetic amplitude mean values.

Next to the evaluation by means of the weighted acceleration and the fourth power vibration dose value, both according to ISO 2631, the signals were analysed regarding their frequency spectrum. The vibration analysis was performed by means of a Fast Fourier Transformation (FFT) to enable the assessment of different frequencies in the spectrum. The different test runs were combined by means of arithmetic amplitude mean values. In many investigated cases there was a distinct frequency of 5 Hz with a significant amplitude (see Fig 8). This was very clear at low tyre pressure (25 kPa). With increasing vehicle speed another peak arises at around 8 Hz. For higher vehicle speed and higher tyre inflation pressure even higher frequencies seem to play an important role in the spectrum.

**Fig 9:**
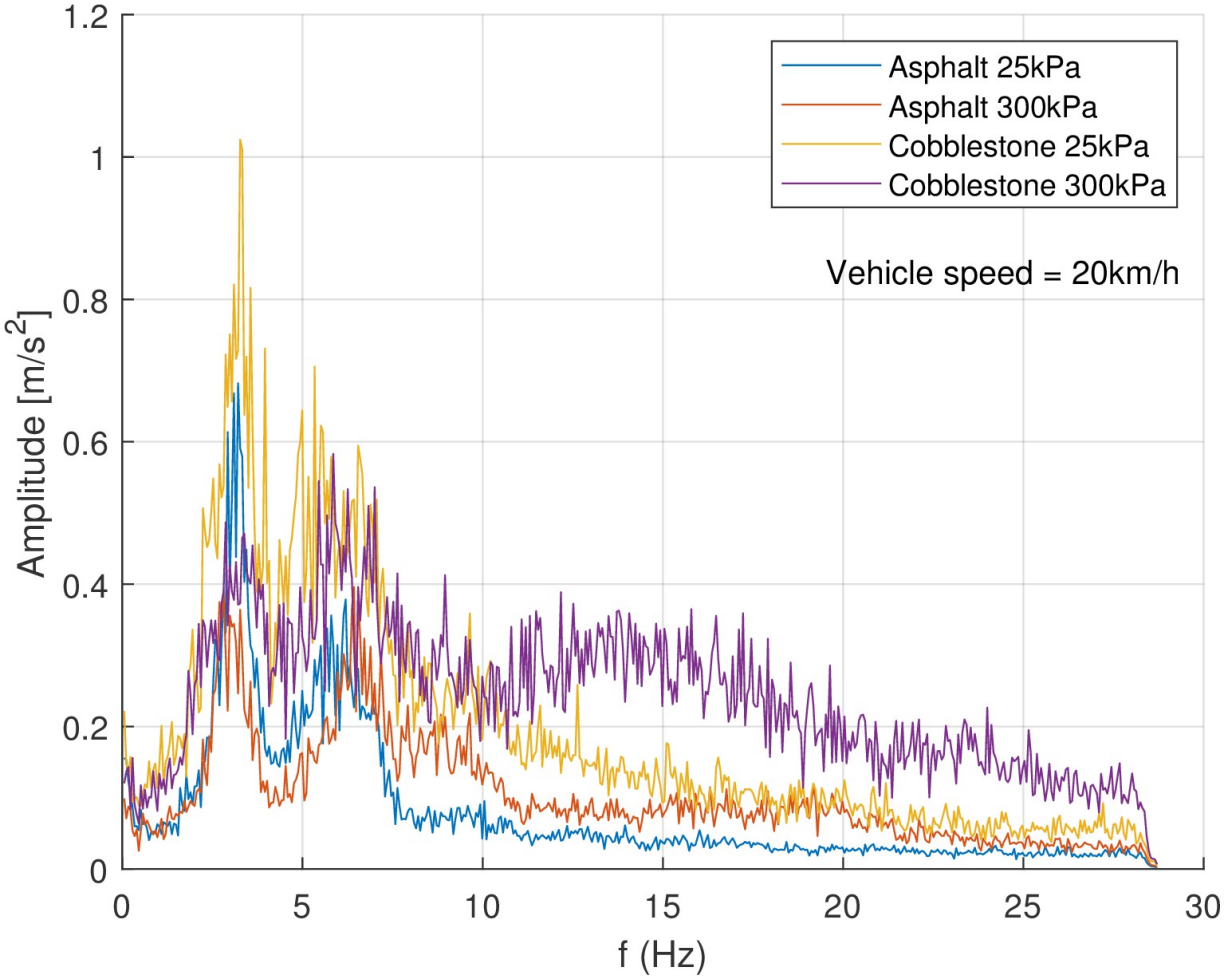
Single sided amplitude spectrum of the vertical acceleration *a*_*z*_ as result of an FFT when driving the bicycle trailer on asphalt and cobblestones. For each setting about ten test runs were combined by means of arithmetic amplitude mean values.

On cobblestones the amplitude was quite high in accordance with the results from the weighted acceleration. However, specifically with low tyre inflation pressure, only around the assumed eigenfrequency of around 5 Hz there was a high amplitude. At higher vehicle speed the amplitude decreased for this frequency but was completed with higher amplitudes at higher frequencies (see Fig 9). The cobblestones had about 8 cm length of edge and gaps of about 2 cm. Depending on the angle of attack, this would lead to a wave length of about 10…14 cm, which is clear in the wavelength of 5…50 cm correlated to driving vibrations as stated by Nilimaa [19]. At a vehicle speed of 10kmh this would result in a frequency of 21…28 Hz if each stone would generate a similar impact. Anyhow, in this frequency range no significant peaks were detected.

## Discussion

The evaluation of the measurements in this work (weighted vibration levels from 1ms2 to 5.5ms2 in extreme cases 9.3ms2) shows that the vibration level in such a bicycle child transport is quite high. With this the results in this study confirm the findings from Schwanitz et al. [20] who found weighted vibration levels in the range between 1.5ms2 and 6.5ms2 where the majority of runs was situated between 3ms2 and 5ms2. However, specifically with the medium tyre inflation pressure of 100 kPa that was assumed to damp the vibration more effective, the vibration level ended up around 9ms2 in this work. Even the RMS values found by van Driessche [21] in the range of 3.5ms2 fit into the here found range of 1.15ms2 to 6.94ms2 completed by two extreme values. However, vad Driessche did not report the tyre inflation pressure used. In contrast, the frequency of the highest peaks in the FFT calculated from van Driessche’s measurements under the head of the dummy, meaning in the child seat, were with 3.4 Hz on cobblestones similar to the measured 3.3 Hz in this work for 25 kPa at 20kmh. The other peaks did not match directly, however in principal 3–4 Hz and 6–8 Hz were common findings of high acceleration.

These similarities also includes the level of vibration in relation to that in automobiles. The unweighted RMS values measured at 30kmh in the car for comparison (1.01ms2 on asphalt and 1.42ms2 on cobblestones) in this work are in the same magnitude as cited by van Driessche [21] (1.44ms2 at 20kmh and 1.88ms2 at 40kmh on cobblestones) and Schwanitz (0.3-2.7ms2).

The results Hölzel et al. [16] found can hardly be compared because of different measurement methods and specifically because of an evaluation according to another standard. (In 2012 when the study was published, the standards VDI 2057 and ISO 2631 differed significantly.) The data of the Ridevibes project [11] are unfortunately not public available. In addition, it is shown here that even at significant lower tyre pressure the vibration level is still quite high. Tyres were expected to take the main suspension work since in the bicycle carrier no chassis suspension was available. Anyhow, the tyres seem to be very stiff. Even at very low tyre inflation pressure of 25 kPa (that increases the risk for damage to tube and rim) the vibration level is still high compared to the measurements in the car. On the cargo-trike the fraction of tyre and suspension was not investigated. One reason for the high tyre stiffness might be that these tyres are made for higher wheel-loads—e. g. recumbent bike front wheels. The maximum load for the tyre is 85 kg. An empty carrier utilised only 9% of the maximum tyre load, a fully loaded carrier utilises 45%. In addition to the lower static wheel load, the load variation might be a challenge: With an empty carrier each tyre has a vertical load of ∼ 8 kg compared to a vertical load of ∼ 38 kg, which results in a factor of 4.75. A challenge that is well-known from comfort issues in heavy trucks. On conventional bicycles as well as in passenger cars the factor between minimum and maximum static vertical load used to be less than 1.5.

Regarding the tests over the artificial step, the correlation between VDV (fourth power vibration dose value according to ISO 2631) and threshold height, tyre inflation pressure as well as vehicle speed are clear (see Fig 7), even if they might be non-linear. However, the results from the outdoor experiments suggested a linear relation, therefore, the steps in-between (100 kPa and 15kmh) were discarded in favour of other experiments.

## Conclusion

The results in this study confirm the findings from Schwanitz et al. [20] and van Driesschen [21] that the vibration level for children in bicycle transportation are significant above them in automotive transportation, and extended them to the range of significant lower tyre pressure, down to 25 kPa, which is far below the recommended tyre inflation pressure level due to risk for damages to the tubes and rims as well as driving risks. However, lower tyre inflation pressure does not correlate directly with lower level of vibration. The experience gained in this investigation suggests that the vertical tyre stiffness has a major impact on the comfort level. In addition, chassis suspension might increase passenger comfort, too. The results of the present study are limited to one type of cycle carrier without chassis suspension, however, the used type of cargo trike with chassis suspension did hardly perform better. Anyhow, Rehnberg and Drugge showed for wheel loaders [4] the potential to reduce the RMS of the combined accelerations from up to 6ms2 down to 1-1.5ms2 by means of chassis suspension.

An investigation about frequency dependent tyre stiffness and the influence of optimised chassis suspension systems on comfort is, therefore, suggested as future work. In addition, the assumption about linearity should be questioned. A wider range of parameters, possibly in a test bed instead of a complete vehicle system, might increase the understanding.

The impact on health of the children inside the cycle carrier is a medical topic that could not be investigated within this study. The ergonomics expert Brecht Daams consulted by van Driessche [21] summarised: The fact that little is known about the effects of vibrations on infants and that there are no standards does not mean that it is not a problem. Therefore, a medical study is recommended as follow-up to understand the impact, specifically the long term effect of such mode of transport.
